# Overview of the BioCreative VI text-mining services for Kinome Curation Track

**DOI:** 10.1093/database/bay104

**Published:** 2018-10-17

**Authors:** Julien Gobeill, Pascale Gaudet, Daniel Dopp, Adam Morrone, Indika Kahanda, Yi-Yu Hsu, Chih-Hsuan Wei, Zhiyong Lu, Patrick Ruch

**Affiliations:** 1 SIB Text Mining, Swiss Institute of Bioinformatics, Geneva, Switzerland; 2 HES-SO / HEG Geneva, Information Sciences, Geneva, Switzerland; 3 University of Kentucky, Lexington, KY, USA; 4 Liberty University, Lynchburg, VA, USA; 5 Montana State University, Bozeman, MT, USA; 6 National Center for Biotechnology Information, Bethesda, MD, USA

## Abstract

The text-mining services for kinome curation track, part of BioCreative VI, proposed a competition to assess the effectiveness of text mining to perform literature triage. The track has exploited an unpublished curated data set from the neXtProt database. This data set contained comprehensive annotations for 300 human protein kinases. For a given protein and a given curation axis [diseases or gene ontology (GO) biological processes], participants’ systems had to identify and rank relevant articles in a collection of 5.2 M MEDLINE citations (task 1) or 530 000 full-text articles (task 2). Explored strategies comprised named-entity recognition and machine-learning frameworks. For that latter approach, participants developed methods to derive a set of negative instances, as the databases typically do not store articles that were judged as irrelevant by curators. The supervised approaches proposed by the participating groups achieved significant improvements compared to the baseline established in a previous study and compared to a basic PubMed search.

## Introduction and motivation

Biomedical big data not only offers tremendous potential for making discoveries but also demands unprecedented efforts to keep structured databases up to date with the findings described in the torrent of publications (1). The neXtProt database (2–3) aims at representing the current state of knowledge on the human proteome. Human curators play a key role in defining the content and ensuring the quality of these reference databases (4–5). Their mission consists of continuously collecting, verifying and annotating the literature. Most curation methods are based on manual approaches, which produce the most accurate knowledge but are also time-consuming (6). At the very first stage of the process, one study (7) estimates that ∼7% of the curation time is assigned to the rejection of papers, while another study (4) assumes that 15% of curators’ time is spent on selecting relevant articles. Triage systems that retrieve, filter and/or prioritize the literature can hence help curators focus on articles appropriate for curation.

The Computer and Laboratory Investigation of Proteins of Human Origin (CALIPHO) group has developed the neXtProt database (3), a flagship resource of the Swiss Institute of Bioinformatics (SIB) that integrates information on human proteins. The data in neXtProt comes from both integration of external resources, and annotation produced within the group using an internal annotation tool, the BioEditor. In a project funded by Merck Serono from 2011 to 2013, the CALIPHO group has annotated 300 human protein kinases from >13 600 research articles, producing a data corpus of >30 000 different annotations describing the kinase functions, their substrates and diseases in which they have been implicated. This large data corpus was still unpublished in 2017 (to be released in 2018), providing a unique opportunity to use an extensive set of curated data for a text-mining competition.

Thus, in 2017, the BioCreative VI Kinome Track proposed a competition in literature triage based on the neXtProt unpublished protein kinase data set. Literature triage is an information retrieval task—it aims at retrieving/filtering articles that are relevant for curation. This is a basic task performed by all virtually curated molecular biology databases to initiate a curation workflow. While this task is usually manually performed, better methods are desirable in order to speed up the workflow. Text-mining groups were invited to develop and test approaches for the selection and ranking of relevant articles for the curation of human protein kinases. The BioCreative VI Kinome Track evaluated triage at two different levels in two different tasks: abstracts triage and full-text triage. While all abstracts annotated in the neXtProt data were available via MEDLINE, the availability of full texts was more problematic, as only a minor fraction (∼10%) was available via open access licensing in services such as Europe PubMed Central (PMC) (8).

## Tasks and data

### The Kinome Track data set

The BioCreative VI Kinome Track data set contains comprehensive annotations about kinase substrates, GO biological processes and diseases. It covers a significant fraction of the human kinome: 300 proteins out of ∼500 human kinases. The data set contains >30 000 annotations. Each annotation is supported by a reference to a publication, a PubMed identifier (PMID). This data set will be integrated in the neXtProt database in 2018, but it was still unpublished at the competition time.

The BioCreative VI Kinome Track focused on two different curation axes: diseases and biological processes. The whole subset represents a total of 4581 curated articles for diseases and 5357 for biological processes. There is a slight overlap between both axes: only 6% of the articles contain both disease and biological process annotations. In total, 9367 different articles, published in 862 different journals, are present in the data set. Table 1 shows the 10 most represented journals.

**Table 1:** Top 10 journals in data set

**Journal**	**No. of articles in data set**	**Cumulative percentage**
*J. Biol. Chem.*	744	7.9%
*Proc. Natl. Acad. Sci. USA*	314	11.3%
*Cancer Res.*	301	14.5%
*Blood*	288	17.6%
*Mol. Cell Biol.*	253	20.3%
*Oncogene*	228	22.7%
*PLoS One*	219	25.1%
*J. Immunol.*	208	27.3%
*Nature*	156	28.9%
*Clin. Cancer Res.*	156	30.6%

* The top 10 journals in the data set, ranked by presence in the data set. The cumulative percentage is computed for the whole collection (e.g. the top 10 journals represent 30.6% of all the annotated articles in the data set).

### The Kinome Track benchmark

In the Cranfield paradigm (9) for evaluation of information retrieval systems, benchmarks are composed of three parts: a collection of documents, a set of queries and relevance judgements. In the BioCreative VI Kinome Track, the query was a human kinase and a curation axis (biological process or diseases). Participants’ systems had to search in a literature collection and produce a ranked list of articles relevant for the query. Finally, systems were evaluated on their ability to rank first the articles that were chosen by the neXtProt expert curators.

#### 

a)* Design of the collection:* For a fair comparison, all systems must be running on a common given collection. For this competition, the collection had to satisfy two conditions: being small enough to be efficiently processed by all teams and large enough to make the task realistic. To reduce the MEDLINE corpus, as the curation was finished in 2013, we filtered out papers published in 2014 and after. The final collection thus contained a total of 5.3 M PMIDs. Unfortunately, for the full-text collection, only a small fraction of the publications (∼10%) was available in open-access licence.

#### 

b) *Queries:* Queries were pairs made of one of the 300 curated kinases (e.g. ACVR1B–P36896), and a curation axis (biological process or diseases). For each kinase, the gene and protein synonyms (extracted from the neXtProt database) were provided to the participants. For example, for the kinase *Mast/stem cell growth factor receptor KIT*, synonyms such as *PBT* or *CD117* were provided to the participants.

#### 

c) *Relevance judgements:* For a given kinase and a given axis, all articles that were chosen by a neXtProt curator—thus present in the data set—were considered relevant. Unfortunately, the rejections of articles after screening by curators are not stored in the database. Thus, the gold standard did not contain non-relevant articles. As systems could submit relevant articles that were never screened by a curator, final performances could be under evaluated. Yet, as these articles are assumed to be equally distributed among all participants’ runs, comparisons between methods are still valid.

### The Kinome Track tasks

**Figure 1:**
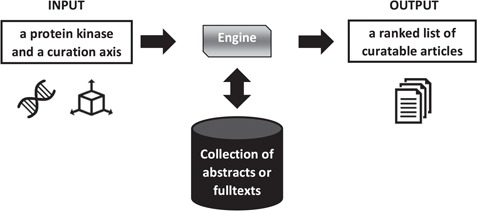
Overview of literature triage for the Kinome Track. The axis is either diseases or biologicial processes. The collection differs depending on the task, abstracts or full texts.

Figure 1 presents an overview of the triage process.

The 300 proteins in the data set were randomly distributed in three different subsets: the tuning set, the task 1 test set and the task 2 test set. The tuning set thus contained 100 kinases, along with the PMIDs of the annotated articles for each axis (relevance judgments). The tuning set was made available in April 2017, and participants were free to use it for analyses and for system tuning. Both test sets also contained 100 kinases and were delivered in May 2017. Obviously, test sets only contained the queries, while relevance judgements were kept for the official evaluation.

#### 

a) *Task 1. abstracts triage:* This task focused on abstracts triage and ranking. The collection (5.3 M citations) was provided in the form of MEDLINE citations. Thus, systems had to perform triage based solely on abstracts and metadata such as journal, publication year, publication type etc. Table 2 provides different statistics about the distribution of papers per kinase in the Kinome Track data set. The collection was provided in BioC format (10).

#### 

b) *Task 2. full-text triage:* This task focused on full-text triage. The collection was given in the form of PMC full texts. Thus, systems had to perform triage based on full-text contents. As only a fraction of PMC is open access, the collection for the task 2 only contained 530 000 articles. Thus, in the test set, for each kinase, this collection contained on average 2.6 relevant articles for the biological process axis and 3.6 for the disease axis. The collection was provided in XML format.

These statistics are summarized in Table 3.

**Table 2:** Distribution of curated papers per kinase

**Axis**	**Quartiles**			**Mean**	**Median**
	**1st**	**2nd**	**3rd**
Disease	7	15	26	21.9	15
GO BP	8	17	28	20.6	17

* Different statistics dealing with the number of curated papers per kinases. The Kinome Track data set contained 300kinases.

**Table 3:** Benchmarks statistics

**Task**	**Collection**	**No. of topics (kinases)**	**No. of relevant papers for disease**	**No. of relevant papers for biol. Proc.**
1. Abstracts	5.3 M abstracts	100	21.9	20.6
2. Full texts	530 000 full texts	100	3.6	2.6

* Statistics for task 1 and task 2 benchmarks. Only 10% of the annotated articles were available via open access licensing, making relevant papers much less numerous in task 2.

### Metrics used for evaluation

Text REtrieval Conference (TREC) formats and metrics (11) were used for evaluation as follows:

- P10 or Precision at rank 10: among the top 10 articles submitted by the system, how many are relevant. If the system submits 10 documents and only 4 are relevant, then P10 is 0.4. P30 and P100 (precision at ranks 30 and 100) were also evaluated.
- R30 or Recall at rank 30: among all the relevant articles in the collection for a given query, how many are retrieved in the top 30 articles submitted by the system. If for a given query there are 20 relevant documents in the collection, and the system submits 10 of them in the top 30 documents, then R30 is 0.5. idem for R100 at rank 100.
- P at R0: maximum precision observed at all ranks.
- Mean average precision (MAP): average of all Precision at rank k, for ranks where a relevant article is retrieved (for queries that have no retrieved articles, 0 is counted).
- R-prec: Precision observed at rank *r*, where *r* is the number of relevant articles for a given query. If for a given query there are 15 relevant articles in the collection, R-prec corresponds to the precision at rank 15.

## Results

More than 20 teams registered to the Kinome Track, and finally two of them submitted results. During the workshop, informal discussions with the various teams revealed that the task was judged as particularly complex. Beyond simple *ad hoc* information retrieval, teams performed information extraction of biological entities (such as proteins, diseases or functions) in order to compute the relevance of documents for triage.

In the following, final participants first describe the strategies they used for their systems. Then, results for both tasks are presented. Each team could submit up to 10 runs for each task. All metrics were computed with the trec_eval reference program (12).

### Participants' strategies

The next two subsections were written by the two participating teams.

#### 

a) *The KinDer system:* KinDER (Kinase Document Extractor and Ranker) is comprised of two main components: (i) Document Retrieval (DR) component, which retrieves documents annotated with kinases and axis terms using dictionaries, and (ii) Document Ranking and Information Extraction (DRIE) component, which uses supervised learning to rank those retrieved documents based on relevancy. For document annotation, the DR component uses ConceptMapper (13) with default settings in conjunction with bio-ontology dictionaries downloaded from National Center for Biomedical Ontology annotator webtool (14). For diseases annotation we used the Human Phenotype Ontology (HPO) (15) and NCITd, which is a hand-culled subset comprised only of disease-related subsections in the National Cancer Institute Thesaurus (16). For biological process annotation, we used the GO. For annotating kinase names, we created a dictionary using the kinase information provided by BioCreative organizers. These kinase dictionaries were expanded by using kinase synonyms from neXtProt and further enhanced by converting Roman numerals to Arabic numerals or removing spaces in the protein name (e.g. from `p145 c-kit’, we build the `p145 c-kit’ synonym, which is present in more than 8300 MEDLINE abstracts).

Following the document annotation above, the DR component performs (i) cross-reference validation, which filters out obviously irrelevant documents that do not contain either the kinase or the axis term; (ii) feature vector generation for the downstream machine-learning model; and (iii) creation of corresponding binary labels based on the BioCreative gold standard. After applying stemming and stop words removal, it generates two types of features: (i) standard Bag of Words (BOW) features, which uses two- and three-gram term combinations, weighted by Term Frequency Inverse Document Frequency (TFIDF) values (17), and (ii) six-engineered (ENG) feature sets. The ENG set contains a kinase score, the number of kinase annotations normalized by total words; Axis Score, the number of axis term annotations normalized by total words; Relevancy Score, the product of the kinase score and axis score; Proximity Score, the minimum number of words separating a kinase and axis annotation; and Proximity 10-Count and Proximity 50-Count, the number of pairs of kinase and axis annotations that are within 10 and 50 words of one another, respectively.

The DRIE component models the task of ranking documents as a binary classification problem in which it distinguishes between relevant and irrelevant articles. We used the Scikit-learn (18) Python machine-learning library for implementing the machine-learning models. For the biological process full text and abstract tasks, eight support vector machine (SVM) models were developed based on SVM kernel (linear vs. Gaussian) and feature type (BOW vs. ENG). For the disease Full Text and Abstract subtasks, 16 models were developed based on kernel (linear vs. Gaussian), features (BOW vs. ENG) and ontology (HPO vs. NCITd). Each classifier model was trained using the full set of gold standard relevant documents and a 10–20% random sample of the total irrelevant documents (depending on balancing strategies). Each trained classifier was used for ranking the test documents based on their relevance according to the classifier confidence scores.

#### 

b) *The NCBI system:* To address the human Kinome Track in BioCreative VI, we formulated the problem as a document classification task where we used rich co-occurrence and linguistic features to prioritize the biomedical articles involving the relations among kinases, diseases and biological processes. Our system is designed for finding and ranking relevant articles for neXtProt human curators in their routine workflow.

Our system consists of automatic bio-concept annotators, feature extractors and machine-learning classifiers. For each article, we first extracted several types of bio-concepts using state-of-the-art Named Entity Recognizer taggers—TaggerOne (19) and tmVar (20, 21)—and integrated their results into the official training set. Since there are no negative training instances provided in the official training set, we generated pseudo-negative data by using a one-class classification method (22). Next, we extracted features such as entity frequency, position and several others for distinguishing relevant vs. irrelevant articles. To tackle the high-dimensional features, we experimented with three different machine-learning algorithms and found that the lasso and elastic-net regularized generalized linear models (23) outperformed the SVMs and convolutional neural networks (24). We find that our system can effectively reduce the workload of human curators and accelerate the workflow of manual curation. For example, as revealed by post-analysis with official gold standard, our system is able to retrieve 5.2 and 6.5 kinase articles with relevant disease and biological process information among its top 100 results, respectively (interpolated from R100 values).

**Figure 2:**
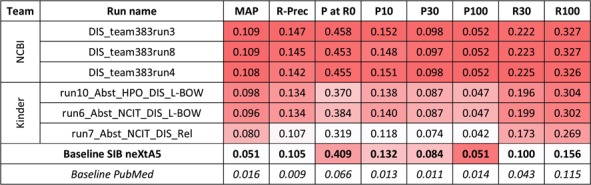
Results for the abstracts triage task, disease axis. The best three runs submitted by each team are presented, along with official baselines. Conditional formating is used for highlighting best participants results, for each metric, in red. The neXtA5 baseline (in bold) is included in the highlighting, while the PubMed baseline (in italic) is not.

### Results of task 1—abstracts triage

In (25), the text-mining group at the SIB describes the development of neXtA5, a curation service and interface, powered by different ontologies and developed for the CALIPHO group. This system aims at assisting SIB curators by prioritizing articles for the curation of a given protein and a given axis. neXtA5 does not perform any machine learning. Entity-named recognition is used in order to detect different entities, such as diseases, GO terms, protein names, species or chemicals. First, a PubMed search is conducted with the kinase name. Then, returned abstracts are parsed, and the density of recognized concepts is used in order to reorder the results and thus to perform triage. Prior to the competition, the neXtA5 system was evaluated on the Kinome Track data set. neXtA5 ranking is also compared with the basic PubMed ranking. These values can be considered as baselines for the interpretation of results in the Kinome Track.

For the first task and the disease axis, 20 runs were submitted. Figure 2 presents the best results for each team, along with performances obtained by the SIB neXtA5 platform, and PubMed, on the same test set. The PubMed result set simply was the output of PubMed when querying with the kinase name; this is how a curator typically queries in his workflow.

Best observed performances are obtained by the National Center for Biotechnology Information (NCBI) submissions. Comparing with the neXtA5 baseline, metrics favoring Recall, such as R30 (+122%) and MAP (+113%), are impressively improved. On the other hand, Precision at high ranks, measured with P at R0 (+12%) and P10 (+15%), are slightly improved. Comparing both participants, we observe that the best KinDer submission is below, yet quite competitive, with the National Center for Biotechnology Information (NCBI) submissions.

**Figure 3:**
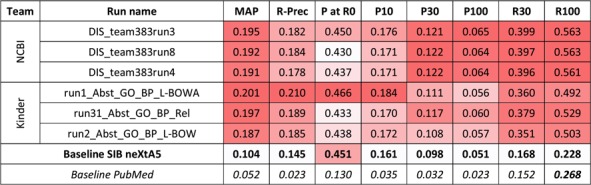
Results for the abstracts triage task, biological process axis. The best three runs submitted by each team are presented, along with official baselines. Conditional formating is used for highlighting best participants results, for each metric, in red. The neXtA5 baseline (in bold) is included in the highlighting, while the PubMed baseline (in italic) is not.

For the first task and the biological process axis, 20 runs were also submitted. Figure 3 presents the best results for each team, along with performances obtained by the SIB neXtA5 platform, and PubMed, on the same test set.

The KinDer submissions seem to be the most performant this time, in particular in high ranks. Comparing with the neXtA5 baseline, observations are similar with the disease axis: metrics favoring recall, such as R30 (+114%) and MAP (+93%), are the most improved, while P at R0 (+3%) and P30 (+13%) are modestly improved. Comparing both axes, the neXtA5 baseline shows better performances with biological processes than diseases: 0.104 vs. 0.051 in MAP (+104%), and 0.451 vs. 0.409 (+10%) in P at R0. The same trend is observed for the submitted runs, in terms of best MAP values (0.201 vs. 0.109, +84%) and P at R0 (0.466 vs. 0.455, +2%).

### Results of task 2—full-text triage

**Figure 4:**
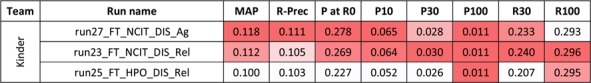
Results for the full-text triage task, disease axis. There was only one submitting team. Conditional formating is used for highlighting best participants results, for each metric.

**Figure 5:**
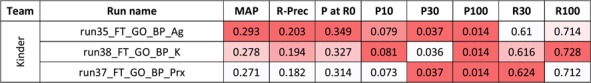
Results for the full-text triage task, biological process axis. There was only one submitting team. Conditional formating is used for highlighting best participants results, for each metric.

For the second task, 10 runs were submitted for the disease axis, and eight for the biological process axis. Figures 4 and 5 present the results for each run. The NCBI team did not submit any run for task 2, and no baseline was available.

As for abstracts, better performances are observed for the curation of biological processes than diseases.Yet, comparisons between abstracts and full-text triage must be done with great care. Indeed, the sizes of the collections render the direct comparison difficult (5.3 M in task 1 vs. 530 000 in task 2). Moreover, the number of relevant articles in the benchmark is 16–18 per query in task 1, vs. 2–3 per query in task 2. In this perspective, the best reported MAP values are higher for full texts than abstracts, but the likelihood of missing some relevant articles in the task 1 is higher. A comparison remains possible with P at R0, since this metric focuses on the precision of the first relevant retrieved article: best reported P at R0 values are higher for abstracts than full texts. P at 0 of 0.349 for the best run means that (on average) one out of the top three results is relevant.

### Task 3—snippets extraction

A third task was initially considered: snippet extraction. In this task, the participants’ system should extract from the full text a snippet of maximum 500 characters, which contains enough information to be `annotatable'. Curators should judge snippets according to one of the three following values: 1 = very good (the snippet is sufficient for making an annotation without reading the paper); 0.5 = acceptable (the curator judges that there is a potential annotation, but needs to read the paper because the snippet is not sufficient for making the entire annotation); and 0 = irrelevant (nothing in the snippet indicates that an annotation is possible).

Several examples made by a SIB curator were provided. For instance, for the kinase MAPK13 in the PMCID PMC4695881, one very good snippet (as chosen by the curator) for disease annotation is `Comparison of DNA sequence reads of PCR products with in silico bisulfite-converted MAPK13 reference sequence (NC_000006.12) identified differential CpG methylation in oesophageal squamous cell carcinoma (Figure 3B)'.

Unfortunately, this task was cancelled due to no submitted runs.

## Discussion and conclusion

Machine learning is currently not considered for the curation of kinase proteins at neXtProt. Initially, when we designed this challenge, we thought that the data set of 300 curated kinases was too small in order to be exploited for such a supervised approach. This is why we only provided 100 kinases in a so-called `tuning’ (and not `training’) set. Moreover, a strong limit of the data set was that it contained only positive instances: articles that were selected for curation. Articles that were scanned but rejected by curators are not stored in neXtProt, thus the data set did not contain any negative instances. Supervised approaches usually need both positive and negative examples in order to learn how to discriminate a new input.

However, both participating groups investigated machine-learning approaches. Pseudo-negative instances were created in order to feed the algorithm. These pseudo-irrelevant articles were selected by teams with random sampling or with One-Class Classification method. Compared with the neXtA5 reference (25), the competing systems showed slightly better performances in the abstracts task in terms of Precision at R0 (+10% for the disease axis), but significant improvements in terms of MAP (+113% for the disease axis and +93% for the biological process axis).

On one hand, these remarkable performances were obtained with only a fraction of available data (100 kinases) used for learning. An optimal design of training and test sets could provide twice as much data for learning, which would probably lead to even better performances. On the other hand, machine learning needs a minimum set of high quality data to begin forming reliable predictions; in this perspective, it is worth emphasizing that the tuning set contained >3300 articles manually selected by human curators. These high quality data sets are neither easy nor cheap to acquire. If supervised approaches are promising for outperforming data-free approaches, they are not suited for new curation tasks. Moreover, each curation task has dedicated scopes, dedicated terminologies and dedicated relevance factors. When high quality data are not available for machine learning, traditional dictionary-based methods are still useful for assisting curators in their literature searches.

To conclude with machine learning and data management, curated databases usually do not store negative examples, such as articles that are screened by the curator but not selected for curation. This is at least partly due to the time-consuming aspect of manual curation and high pressure from the databases to produce as much output as possible. Paradoxically, this loss of information impedes potential progress by automated tools to speed up the curation process. Hopefully, such a data stewardship `gap’ should improve as FAIR principles become common practice (26).

## Notes

**Database URL:** https://www.nextprot.org/
